# 5-HTTLPR and Early Childhood Adversities Moderate Cognitive and Emotional Processing in Adolescence

**DOI:** 10.1371/journal.pone.0048482

**Published:** 2012-11-28

**Authors:** Matthew Owens, Ian M. Goodyer, Paul Wilkinson, Anupam Bhardwaj, Rosemary Abbott, Tim Croudace, Valerie Dunn, Peter B. Jones, Nicholas D. Walsh, Maria Ban, Barbara J. Sahakian

**Affiliations:** 1 Development and Lifecourse Research Group, Department of Psychiatry, University of Cambridge, Cambridge, United Kingdom; 2 The Cambridge and Peterborough National Health Service (NHS) Foundation Trust, Cambridge, United Kingdom; 3 Department of Clinical Neurosciences, University of Cambridge, Cambridge, United Kingdom; 4 MRC/Wellcome Trust Behavioural and Clinical Neuroscience Institute, University of Cambridge, Cambridge, United Kingdom; University of Sydney, Australia

## Abstract

**Background:**

Polymorphisms in the promoter region of the serotonin transporter gene (*5-HTTLPR*) and exposure to early childhood adversities (CA) are independently associated with individual differences in cognitive and emotional processing. Whether these two factors interact to influence cognitive and emotional processing is not known.

**Methodology and Principal Findings:**

We used a sample of 238 adolescents from a community study characterised by the presence of the short allele of *5-HTTLPR* (LL, LS, SS) and the presence or absence of exposure to CA before 6 years of age. We measured cognitive and emotional processing using a set of neuropsychological tasks selected predominantly from the CANTAB® battery. We found that adolescents homozygous for the short allele (SS) of *5-HTTLPR* and exposed to CA were worse at classifying negative and neutral stimuli and made more errors in response to ambiguous negative feedback. In addition, cognitive and emotional processing deficits were associated with diagnoses of anxiety and/or depressions.

**Conclusion and Significance:**

Cognitive and emotional processing deficits may act as a transdiagnostic intermediate marker for anxiety and depressive disorders in genetically susceptible individuals exposed to CA.

## Introduction

Allelic variation in the promoter region of the serotonin transporter (*5-HTTLPR*), encoded by a single gene (SLC6A4), is partly responsible for regulating serotonergic (5-hydroxytryptamine, 5-HT) functions in the brain [1]. *5-HTTLPR* has been considered functionally bi-allelic, where the short (S) allele of this region is associated with lower transcriptional activity, less 5-HT uptake, binding and lower concentrations [2], [3]. An in vivo study also showed higher rates of 5-HT uptake in platelets for the long (L) compared to the S allele [4]. More recently, research has suggested that an A>G single-nucleotide polymorphism (SNP; rs25531) makes the L allele function similar to the S allele when it is L_G_ rather than L_A_ [5], [6], [7], although this literature is somewhat inconsistent [8], [9]. Human brain imaging (PET) findings also suggest that if there are differences they may occur more in moderating neurogenesis or methylation processes than binding as such but the precise mechanisms remain somewhat unclear [9]. Two studies have shown that although the L_G_ and S alleles are functionally similar, the S and L_A_ alleles displayed the lowest and highest functioning, respectively, in absolute terms [7], [10]. Originally postulated to play a causal role in the development of anxiety-related traits [3] or affective disorder [11], subsequent research has suggested that *5-HTTLPR* has a moderating role in the presence of other factors such as an adverse social environment [12].

The interplay between genes and environment in psychopathology is comprised of two broad categories: gene-environment interactions (G×E) and gene-environment correlations (rGE) [13], [14], [15]. In the seminal G×E interaction study on depression by Caspi et al. [16] the probability of a major depressive episode in young adulthood was approximately doubled for S allele homozygotes when compared to their L allele homozygote counterparts, among people with multiple recent environmental adversities. This G×E effect was subsequently replicated in maltreated and bullied children [17], [18]. Following the work of Caspi and colleagues [16] there has been a considerable amount of research testing for the presence of G×E with both positive and null effects being reported. Compared to previous publications [19], [20] the largest meta-analysis to date has supported the G×E hypothesis indicating a diathesis-stress model involving some form of within person vulnerability for subsequent clinical affective disorders [21]. Further theoretical accounts, however, have proposed a broader impact for common gene variants, such as present in *5-HTTLPR*, conferring differential susceptibility to the social environment [22], [23] or a biological sensitivity to context [24], [25]. Thus, rather than being specifically ‘vulnerable’ to negative environmental risks, these views suggest that individuals with the S allele are more susceptible or sensitive to the environment in general; whether it is ‘good’ or ‘bad’. Accordingly given a positive environment, individuals homozygous for the S allele (SS) may have significantly lower levels of emotional difficulties and symptoms than their LL counterparts. Experimental study has yet, however, to reveal the precise nature of the emotional bias in S carriers. For example *5-HTTLPR* S carriers can demonstrate a significant attentional bias toward negative or threatening stimuli such as words [26] or spiders [27]. Equally, however, attentional biases towards emotionally positive as well as negative images occur in S carriers [28], [29]. Other studies have shown that S carriers display a greater bias towards negative stimuli (angry faces) while L carriers show the reverse bias, towards positive stimuli [30]. Finally a recent report showed that strong attentional biases can be trained in S carriers for both positive and negative stimuli [31].

There has been increasing interest in how both genes and the social environment are related to cognitive and emotional processing that is associated with affective disorders [32]. Recent conceptual models of emotion psychopathology [33] suggest that cognitive abnormalities such as a negative affective bias may be a product of genetic polymorphisms (e.g. *5-HTTLPR*) and environmental influences, while also being a central cause of the development of emotional disorder. Whether the translation of the G×E effect into disorder is likely to be due to cognitive and emotional information processing is a testable neuropsychological hypothesis.

Studies across the lifecourse have demonstrated that being an S allele carrier is related to differential cognitive and neural processing of emotion stimuli. As well as attentional bias [26], [34], [35] S carriers show increased amygdale reactivity in adolescent anxiety and depression [36], errors made in emotion classification in both typically developing children and ecstasy users [37], [38] and errors on tasks in response to negative feedback [39], [40]. Similarly, exposure to adverse family environments, including parental discord and various forms of privation and neglect, are frequently unpredictable and evoke high emotional sensitivity, maladaptive cognitive distortions and emotion processing biases in offspring [41], [42], [43]. While these studies addressing the independent effects of *5-HTTLPR* and childhood adversities (CA) have revealed much about how variation in these factors can moderate emotional and behavioural responses, they do not take into account any *5-HTTLPR*×CA interactive effects that may exist. Indeed some have argued that main effects research has now reached a critical mass and that gene-environment interplay accounts should take more prominence [32].

A range of studies in children and adolescents has shown that deficits in cognitive and emotion processing are fundamental to common mental disorders, such as anxiety and depression, in this age range [44] and can predict the emergence of future emotional symptoms [45]. In addition studies with adults have shown increased errors in response to negative feedback on task performance (termed a ‘catastrophic response to perceived failure’) [46], [47] in depressed patients compared to well controls. This latter observation has led to the speculation that catastrophic response to feedback may be fundamental to the cognitive architecture of clinical depression [48], [49]. Determining the origins of individual differences in such information processing may therefore extend our understanding of which individuals are at risk for mental illness by revealing intermediate phenotypes that are genetically and environmentally determined.

A recent study focusing on proximal stressful life events and *5-HTTLPR* found no evidence for an interaction on current and recurrent depressions [50]. This leaves open the possibility that exposure to family adversities in early childhood could be driving the effect in genetically susceptible individuals. One major methodological imperative is to establish with the best retrospective interview methods available the quality and quantity of exposure to adversities in the childhood years [51], [52].

In this study we used selected data from a new semi-structured interview-based assessment [53] that collated exposure to the early family environment of adolescents and assessed the interactive effects with *5-HTTLPR* on self-reported anxiety and depression symptoms. We have reported that in the cohort from which this sub sample is drawn exposure to an adverse family environment over childhood and early adolescent years (up to 14) is associated with an increased risk for common adolescent emotional and behavioural psychopathologies with odds ratios ranging from 2.0–8.0 [53].

In the present study, individual differences in cognitive and emotional processing including attentional bias and response to negative feedback are hypothesised as arising from the product of the combined effects of exposure to CA and possessing two S alleles in *5-HTTLPR*. We tested the specific hypothesis that cognitive and emotional neuropsychological deficits will be revealed in adolescents exposed to CA up to the age of 6 who are homozygous for the S allele in *5-HTTLPR*. In order to achieve our objective, we compared performance on tasks that draw on attentional bias, response to negative feedback, and simple visuo-spatial memory in a sub-sample taking part in a longitudinal study of adolescent development.

## Methods

### Participants

Participants were sampled from a larger cohort of individuals (the ROOTS project) recruited from secondary schools in Cambridgeshire and Suffolk, UK [54]. In the present embedded study, an opportunity sampling method was used to identify from available adolescents (around 800 at the time of this sub study) in the ROOTS cohort an initial sample of 277 (aged 15–18), selected from the database on the basis of the 2 factors of interest: *5-HTTLPR* (LL,LS,SS) and exposure to CA (presence/absence) before the age of 6 years to elicit a multi-group design. Participants were subsequently excluded if the database showed them to be of non-Caucasian ethnicity (self-report; n = 25), or had a diagnosis of attention deficit hyperactivity disorder or a neurodevelopmental disorder (n = 6). At assessment a further 5 were excluded because they had an IQ lower than 70 (n = 4), or were intoxicated with alcohol or drugs (n = 1). Participants with available genetic and environmental data (n = 238 of 241) were then classified by bi-allelic variants within the promoter region of *5-HTTLPR* into 3 groups (LL, LS, SS) and exposure to CA up to 6 years of age. The latter had been previously ascertained blind to genetic status and future cognitive and emotional test performance. Those who were both SS carriers and exposed to CA constituted the ‘at risk’ group.

Given the uncertainty over the functionality of the A>G SNP associated with *5-HTTLPR* in the human brain [9] we carried out follow-up analyses, re-classifying participants according to the triallelic genotyping following Parsey et al. [9]. The prime symbol used indicates a change from the original metric: L_A_L_A_ were classified as L′L′; L_A_L_G_ & L_A_S were classified as L′ S′; L_G_ S & SS were classified as S′S′. Additionally, we re-analysed the data using the L_A_ allele only (L_A_L_A_ = L″L″; L_A_S = L″S″; SS = S″S″) to focus on the distinction between the highest (L_A_) and lowest (S) variations considered involved in transcriptional activity [10]. The protocol and procedures for the study were all carried out in accordance with the principles outlined in the Helsinki Declaration [55]. Written informed consent was obtained from all participants and their parents in compliance with the Cambridgeshire 2 REC local ethics committee (reference number 03/302).

### Materials

#### The Cambridge early experiences interview

CA occurring before the age of 6 was measured using the Cambridge Early Experiences Interview (CAMEEI) [53] which is conducted with the child's primary care-giver, predominantly biological mothers (96%) in the ROOTS study. To assess inter-rater agreement on the CAMEEI, 48 interviews were observed by a 2^nd^ trained interviewer and the responses independently double coded. Agreement was high (kappa = 0.7–0.9) on those core indicators with sufficient positive endorsements to permit analysis (any family discord, parenting and any financial difficulties). In the present study, we focused on any episodes of family discord of sufficient severity to impact on family functioning and any incidents of abuse (physical, sexual or emotional). There were no incidents of sexual abuse reported at this age in this subsample and physical or emotional abuse never occurred in the absence of family discord. Reported family discord ranged from mild (e.g. constant bickering) to moderate (e.g. shouting, throwing things, a complete breakdown in communication between family members) to severe (e.g. domestic violence) and negatively impacted family functioning in each case. Adolescents were then classified into those exposed and not exposed to CA.

#### Self-Reported Anxiety and Depression Symptoms

All participants completed the Mood and Feelings Questionnaire (MFQ) [56] at the time of testing. The MFQ has good psychometric properties [57], [58] and also established criterion validity as a screen for adolescents with unipolar depression [59]. Self-report anxiety data were also available for participants, collected as part of the wider ROOTS study in the form of the Revised Children's Manifest Anxiety Scale (RCMAS) [56]. The RCMAS is a 28 item instrument with established reliability in school age children that measures general anxiety, including physiological anxiety, worry/oversensitivity, and social concerns.

#### Diagnostic Assessment for Psychopathology

All participants were assessed for DSM-IV current anxiety, depression or dysthymia disorders (herein emotional disorders) at 17 years, with the K-SADS-PL [60], also collected as part of the overall ROOTS study. We included participants with sub-threshold conditions who had 3 or 4 symptoms but with overt psychosocial impairment.

Both the diagnostic interviews and self-report anxiety measurement took place before and after testing in the present sub-study (mean time elapsed = +1.02 years, SD = .57, range = −1.14 to 1.84).

#### Genotyping

DNA from saliva samples (Qiagen, Crawley, UK) was genotyped for allelic variation in *5-HTTLPR*. This region was amplified using the primers 5′-ATGCCAGCACCTAACCCCTAATGT- 3′ and 5′- GGACCGCAAGGTGGGCGGGA-3′ which generate a 419 bp and 375 bp product for the ‘L’ and ‘S’ alleles, respectively. The polymerase chain reaction mixture consisted of: 100 ng genomic DNA, 10 mM. Tris-HCl (pH 9.0), 1.5 mM MgCl2, 50 mM KCl, 0.1% Triton1X-100, 1.25 U Taq DNA polymerase, 200 mM dNTPs, 500 nM each of forward and reverse primer and 100 mM 7-Deaza-dGTP in a final reaction volume of 15 µL. The reaction conditions were 98°C for 7 min, followed by 40 cycles of 96°C for 30 secs, 61°C for 30 secs and 72°C for 1 min with a final extension stage of 72°C for 10 mins. Polymerase chain reaction products were electrophoresed on a 3700 DNA analyser (Applied Biosystems) with semi-automated sizing and genotyping performed using GENESCAN v3.7 and GENOTYPER v3.7 software for Windows (Applied Biosystems).

Triallelic genotyping was performed using Taqman methodology on a 7900 Sequence Detection System (Applied Biosystems). A 181 bp fragment was amplified using the primers 5′-GCAACCTCCCAGCAACTCCCTGTA-3′ and 5′-GAGGTGCAGGGGGATGCTGGAA-3′. Each reaction contained two fluorogenic probes that are specific for the L_A_ allele (5′-6FAM-CCCCCCTGCACCCCCAGCATCCC-3′) and the L_G_ allele (5′-VIC- CCCCTGCACCCCCGGCATCCCC-3′). PCR amplification of the DNA was completed using 50 ng DNA, 1× Taqman Universal Mastermix (Applied Biosystems), 500 nM each of forward and reverse primer, 80 nM FAM probe (L_A_ allele) and 100 nM VIC probe (L_G_ allele) in a final reaction volume of 5 µL. PCR amplification conditions were 96°C for 10 mins followed by 40 cycles of 96°C for 15 secs and 69°C for 1 min. Following PCR amplification, an end-point reading of the fluorescence from each probe was measured, with the relative fluorescence of each probe used to genotype individuals. Genotyping was completed using the Sequence Detection System Software Version 2.1 (Applied Biosystems).

#### Neuropsychological Assessment

**Figure 1:**
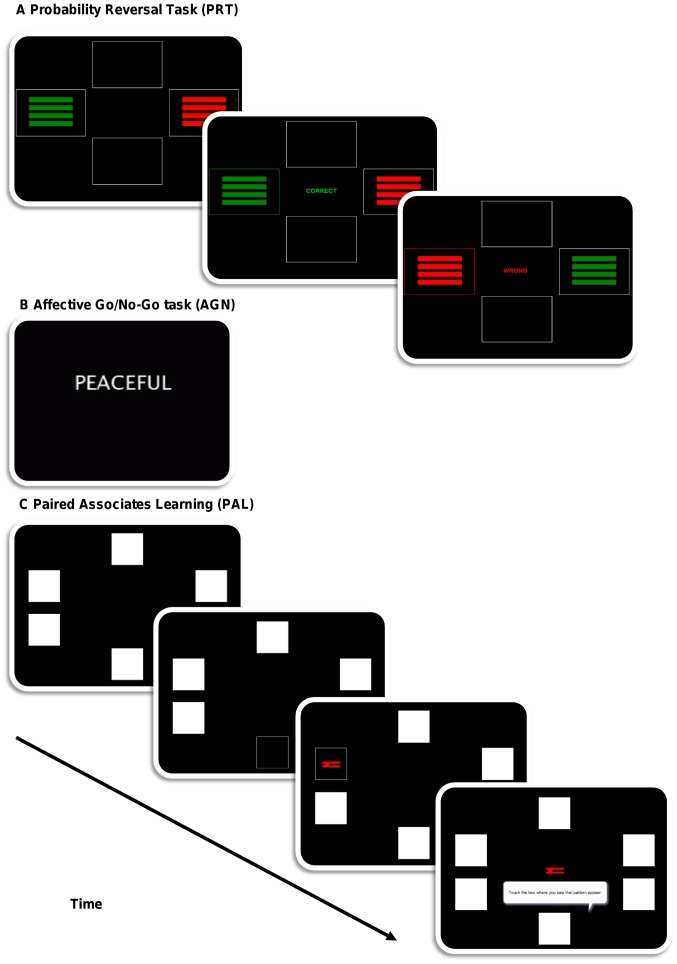
A schematic representation of the computerised cognitive and emotional tasks. Figure 1a shows a representation of the Probability Reversal Task (PRT). Two stimuli are initially presented on the screen. The participant is asked to select one of the stimuli and the feedback “CORRECT” is given. In 20% of trials, participants are given negative reinforcement (“WRONG”). Figure 1b shows a word from the positive condition of the Affective Go/No-Go (AGN) task. Participants are asked to respond with a button press when they see a target word as fast as they can without making mistakes. Figure 1c shows the Paired Associates Learning task (PAL), in which six boxes are opened in a randomised order. A geometric shape(s) appears in one of the boxes. After all boxes are opened the geometric shapes appears in the center of the screen and the participant must select the box in which the shape appeared.

Participants completed the Probabilistic Reversal Task (PRT) and the following two tasks from the Cambridge Neuropsychological Test Automated Battery, CANTAB® [61]; the Affective Go/No-Go task (AGN) and the Paired Associates Learning (PAL), control task. These tasks were delivered via a portable touch screen computer. A schematic representation of the tasks is shown in Figure 1.

#### Probability Reversal Task (PRT)

The PRT [48] measures response to negative feedback. The task requires participants to learn a visual discrimination between two stimuli (stage 1) and subsequent reversal of this discrimination (stage 2). On each trial, two stimuli composed of either four red or four green horizontal lines are presented on the screen. Participants must choose one of the two coloured stimuli by touching it. The first touched by the participant in the discrimination stage of the task is the ‘correct’ stimulus for that stage and receives an 80∶20 ratio of positive/negative reinforcement, presented in both visual (‘correct’ or ‘wrong’) and auditory (a high or low tone) forms. Instructions to the participants were given as follows: “*On each go, the same two patterns will be presented. One of the patterns is correct and the other pattern is wrong and you have to choose the correct pattern on each go.”* Participants were instructed to continue with the stimulus that was usually correct, even if it was occasionally wrong. *“However on some goes, the computer will tell you that you were wrong even if you chose the correct pattern. Your task is to stick to the pattern that is usually correct. So in other words always choose the pattern that is correct more often than the other pattern.”* Not continuing with the ‘correct’ stimulus is referred to as ‘switching’. In the subsequent reversal stage, the previously ‘incorrect’ stimulus becomes the correct stimulus (in this example the green colour). Success criterion on the task is met after eight successive correct answers. We used the total number of errors made before reaching criterion and the probability of switching as dependent variables for this task.

#### Affective Go/No-Go (AGN)

Attentional bias was tested using the computerised Affective Go/No-Go (AGN) task from the CANTAB® battery [62], [63]. The task requires participants to respond to a happy, sad or neutral word in 20 blocks and involves judging the emotional tone of the word stimuli. Half the words are the target valence (e.g., happy), half are the distractor valence (e.g. sad or neutral). Participants must press the button only when they see a word of the target valence. The instructions given were as follows: “*All you have to do is press the button as fast as you can as soon as you see a [target valence] word. Remember to respond as fast as you can, whilst trying not to make any mistakes.”* The words in each target are matched for word length and frequency. Examples include “joyful” for positive; “failure” for negative; and “range” for neutral. After two practice blocks, there are 18 test blocks balanced for valence of targets/distractors. Commission errors (failure to ignore distractors) were used as dependent variables. The task has previously been used with adolescents but without the neutrally valenced words [62].

#### Paired Associates Learning (PAL)

This test assesses simple visuo-spatial memory ability which is in contrast to the AGN and PRT in that there is no overt emotional content in the task. White boxes are displayed on the screen and automatically opened in a randomised order revealing either a geometric pattern or a blank space. After all boxes are opened in a given trial, each pattern is subsequently displayed in succession in the centre of the screen and participants are asked to touch the box where they think the pattern was originally displayed. Each stage (1–6) can include up to 10 trials. If participants get all locations correct, they proceed to the next stage which includes an additional geometric shape to remember. If the location is not correctly identified (up to 9 further attempts are allowed), the test terminates. Participants were given the following instructions: *“In this test you will see six white boxes and they will open up in a random order. There will be a pattern in [stage number] of the boxes. You have to remember which pattern is in which box.”* The total number of errors was used as the dependent variable.

### IQ

#### Wechsler Intelligence Scale for Children-III (WISC)

IQ was estimated using a short form dyad (block design and vocabulary) of the Wechsler Intelligence Scale for Children-III [64]. These scales have been validated for use outside the full assessment [65] and ultimately combine to represent a prorated full-scale IQ score.

### Analysis

The distributions in the data were inspected and where appropriate square root transformations were applied (MFQ, RCMAS and AGN) in order to better align the data with normal distributions (see Table S1). In all other cases (PRT and PAL), variables followed a negative-binomial distribution and were treated as count data.

We first explored the previously reported [66] *5-HTTLPR*×CA interaction on current depressive symptoms. We also conducted this analysis with anxiety symptoms to test for specificity of any effects. We then moved to testing *5-HTTLPR*×CA interactions on the cognitive and emotional variables. To test the *5-HTTLPR*×CA interaction hypothesis, multigroup modelling was employed using genetic status as the grouping variable and allowing CA to predict the self-report mood and the cognitive and emotional variables in each gene group. These analyses were carried out in the Mplus programme (version 6.1).

In our multigroup modelling, an interaction is tested for by comparing an unconstrained model, where CA is freely associated with the dependent variables, against a more restricted (nested) model that constrains the regression coefficients between CA and dependent variables to be equal in all three gene groups. Our *null* hypothesis, therefore, was that the associations between CA and the dependent variables in the LL, LS and SS gene groups would be equal. This tested for no meaningful difference in how CA was related to mood or cognitive variables across the gene groups. A difference between the two models provides evidence for an interaction. For normally distributed variables (MFQ, RCMAS and AGN), linear regression models were specified; negative binomial regression was used for count data variables (PRT and PAL). To formally test for these model differences, we used a Δχ^2^ difference test in the normal linear regression models and the Wald test in the count data models to compare the effects of CA in each gene group. The former is used where χ^2^ for model fit was available. In the latter set of models, χ^2^ is not available so an equivalent method using the Wald test was used.

A significant χ^2^ or Wald test, therefore, suggested the presence of a *5-HTTLPR*×CA interaction effect. The significant interactions were decomposed by assessing the relationship between CA and the dependent variables in each genotype group. As a correction for multiple comparisons in these follow up tests we used the sequentially rejective multiple test procedure described by Holm [67], which is similar but a more powerful alternative to the Bonferroni method [68]. This technique applies a sequential correction of α/n for the highest ranked *p*-value in a set of tests, moving to α/n-1 until α/1 is reached. The procedure is stopped at any point when there is a failure to reject the null hypothesis. For 3 gene groups the following levels of alpha were therefore used: α<0.016, followed by α<0.025 and α<0.05.

A maximum likelihood mean and variance adjusted estimator (MLMV) was chosen for our linear multigroup models which is suitable for use with a priori groupings of different size [69] and is also robust to non-normality [70]. Cohen's *ƒ^2^* effect sizes are given for the overall linear regression models using the following formula *ƒ^2^* = *R* ^2^/1- *R* ^2^. Confidence intervals for *ƒ^2^* were computed using an online calculator [71]. Cohen gives the following guide to estimating the magnitudes of these effect sizes:.02 = small; .15 = medium; ≥.35 = large [72]. For the negative binomial regression models, *R^2^* was not available and so the incidence rate ratio (IRR) was used as an alternative, which is analogous to an odds ratio and is computed by finding the exponent of the regression coefficient. In this way we estimate the difference in rate of task error (PAL and PRT) and probability of switching (PRT) by independent groups (*5-HTTLPR* and CA).

Given the known sex difference on reported mood and diagnosis of an emotional disorder, effects on these variables were adjusted for sex. That is, the binary variable sex was included in these models as a predictor. In order to assess the potential for the cognitive and emotional variables to act as intermediate markers between *5-HTTLPR*×CA interactions and psychopathology, per se, we assessed their association with the presence or absence of an emotional disorder. To explore this relationship, we used logistic regression models where the reference group included individuals with no disorder. The Odds Ratios (OR) and 95%CIs were adjusted for sex.

Thus our data analytic strategy was to test for:

- A *5-HTTLPR*×CA effect on depressive and anxiety symptoms to assess for specific vulnerability for emotional symptoms.
- A *5-HTTLPR*×CA effect on attentional bias for negative words and a deleterious response to negative feedback with no effect on memory functions.
- A significant association between neuropsychological deficits and the probability of receiving a diagnosis of an emotional disorder.

We expected SS carriers to report more emotional symptoms and commit more errors on the cognitive and emotional computerised tasks given a negative early environment (CA). Whereas we expected LL carriers to be less affected within a similar negative environment (CA). In exploratory secondary analyses, we tested the effect of removing the L_G_ allele or reallocating it to form bi-allelic groups, after Parsey et al. [9]. We have taken these 2 approaches because previous human in vivo imaging studies do not agree on the effect of the rs25531 SNP on expression of serotonin transporter [9]. Although there are in vitro studies reporting differential expression of mRNA by the rs25531 SNP [7], [10], some studies have not supported this finding [8]. In addition, the results from the in vitro studies mentioned above suggest that the L and S alleles are the highest and lowest functioning of all alleles, in absolute terms. Therefore we test a triallelic interaction model and one where the L_A_ and S alleles only are included.

## Results

The *5-HTTLPR* genotypes were distributed according to Hardy-Weinberg equilibrium: LL (n = 87; 36.6%), LS (n = 107; 44.9%), SS (n = 44; 18.5%); χ^2^ = 1.18, df = 1, *p* = 0.28. This distribution was not statistically different from that in the overall ROOTS sample (LL = n = 352; 30.3%; LS = n = 596; 51.3%; SS = n = 214; 18.4%), χ^2^ = 3.99, df = 2, *p* = .14. There were also equal numbers of males and females in the test sample (females = 121; 50%; males = 120; 50%) a distribution which was not significantly different to the overall ROOTS sample (females = 674; 54%; males = 564; 46%; χ^2^ = 1.46, df = 1, *p* = 0.23). In order to rule out gene-environment interplay in the data other than an interaction [14], [73] we first tested for a gene-environment correlation (rGE). There was no association between *5-HTTLPR* and maternal reports of exposure to adversity (polychoric *r* = .02, *p* = 0.86). The sample characteristics by genotype and CA are shown in Table S2 and the cognitive and emotional scores in Table 1. Correlations between study variables are shown in Table 2. IQ was consistently and negatively correlated with the cognitive and emotional neuropsychological test variables and was therefore included as a covariate in following analyses involving these variables.

**Table 1:** Cognitive and emotional test scores by 5-HTTLPR and CA groups.

	LL				LS				SS			
	N = 87				N = 107				N = 44			
	CA		No CA		CA		No CA		CA		No CA	
AGN positive	11	(5.6)	9.6	(7.6)	9.3	(6.9)	9.2	(6.1)	11.4	(7.5)	7.4	(5.2)
AGN neutral	14.3	(5.5)	14.5	(7.1)	13.4	(7.7)	13.7	(6.2)	17.1	(7.7)	11.4	(4.7)
AGN negative	10.1	(5.2)	9.2	(7.4)	7.8	(6.8)	8.6	(5.9)	11.8	(8.6)	6.8	(6.1)
PRT s1 errors	1.4	(2.7)	1.4	(2.7)	1.4	(3.2)	1.6	(3.9)	4.9	(5.2)	0.6	(1.4)
PRT s2 errors	6.3	(4.5)	3.9	(2.0)	6.0	(5.0)	5.9	(5.1)	4.0	(5.0)	8.6	(6.9)
PRT s1 switching	0.7	(1.4)	0.6	(1.1)	0.5	(1.1)	0.6	(1.0)	1.3	(1.5)	0.3	(0.6)
PRT s2 switching	1.2	(1.6)	0.7	(0.8)	0.6	(0.8)	0.6	(0.9)	1.4	(1.8)	0.3	(0.5)
PAL errors	6.7	(7.9)	7.9	(9.1)	6.7	(5.3)	6.6	(8.0)	6.9	(7.1)	6.6	(4.2)

*Note.* AGN = Affective Go/No-Go; PRT = Probability reversal task (stage 1&2); PAL = Paired associates learning. In the statistical analyses the PRT and PAL variables were analysed using a negative binomial approach to the data, and not the mean scores.

**Table 2:** Correlation matrix for study variables.

	1.	2.	3.	4.	5.	6.	7.	8.	9.	10.
1.IQ										
2.MFQ	−0.09									
3.RCMAS	−0.02	0.55**								
4.AGN positive	−0.36**	0.07	−0.03							
5.AGN neutral	−0.27**	0.17*	0.08	0.74**						
6.AGN negative	−0.36**	0.08	0.01	0.82**	0.75**					
7.PRT s1 errors	−0.21**	0.01	0.07	0.17*	0.22**	0.17*				
8.PRT s2 errors	−0.18*	−0.03	0.03	0.26**	0.24**	0.24**	0.48**			
9.PRT s1 switching	−0.22**	0.07	0.10	0.10	0.11	0.12	0.66**	0.40**		
10.PRT s2 switching	−0.25**	0.08	0.07	0.23**	0.22**	0.24**	0.43**	0.55**	0.57**	
11.PAL errors	−0.30**	−0.07	−0.12	0.28**	0.13*	0.21**	0.07	0.10	0.06	0.03

*Note.*

* <.05;

** <.01 IQ = Weschler intelligence scale for children (III); MFQ = Mood and Feelings Questionnaire; RCMAS = Revised Children's Manifest Anxiety Scale; AGN = Affective Go/No-Go; PRT = Probability reversal task (stage 1/2); PAL = Paired associates learning.

### Symptoms of Depression and Anxiety

**Figure 2:**
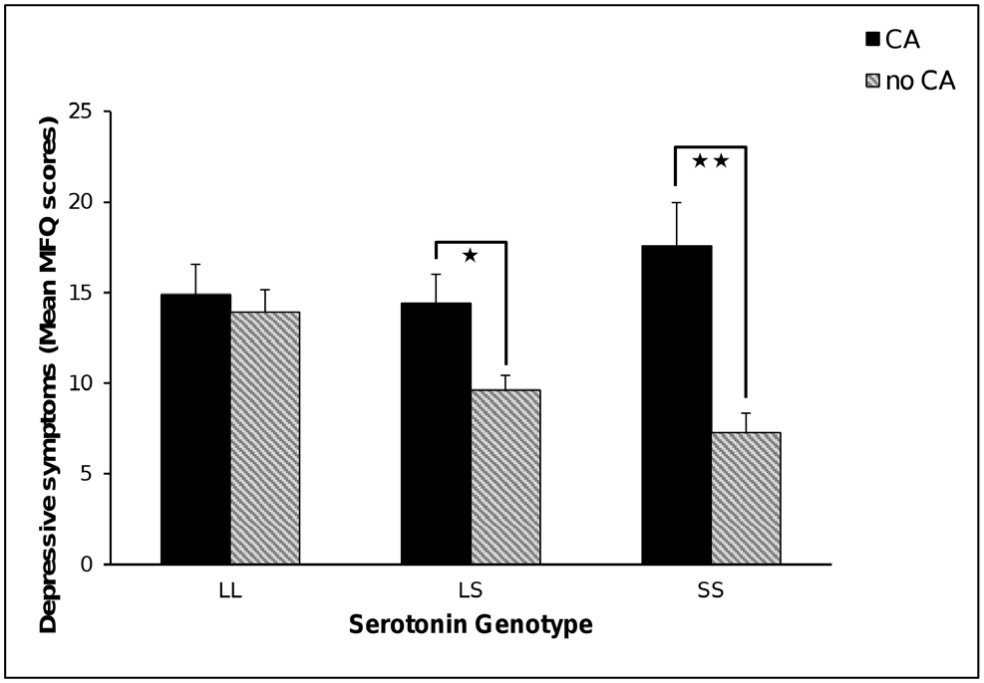
Mean scores of self-reported depressive symptoms by gene variants in *5-HTTLPR* and early childhood adversities (CA). Error bars represent ±1 SE of the mean. * *p*<0.05; ** *p*<0.001.

There were significant main effects with the presence of CA being associated with higher MFQ scores (B = .63, SE = .17, *p* = 0.0001, *ƒ^2^* = 0.06, 95%CI 0.00 to 0.13). There was also a significant main effect of being a *5-HTTLPR* S carrier with lower MFQ scores (B = −.51, SE = .23, *p* = 0.026, *ƒ^2^* = 0.03, 95%CI −0.01to 0.08). This main effect for the S allele was explained by a significant *5-HTTLPR*×CA interaction (Δχ^2^ = 12.59, df = 2, *p* = 0.002). The interaction was decomposed by assessing the relationship between CA and MFQ in each gene group. Follow up comparisons using the Holm correction showed that CA was significantly related to higher depressive symptoms for S carriers in both the LS (B = .71, SE = .25, *p* = 0.004, *ƒ^2^* = 0.13, 95%CI 0.00 to 0.29) and SS (B = 1.49, SE = .33, *p*<0.0001, *ƒ^2^* = 0.55, 95%CI 0.17 to 1.30) groups. In contrast, there was no impact of CA on depressive symptoms in the LL group (B = −0.01, SE = .27, *p* = 0.961, *ƒ^2^* = 0.17, 95%CI 0.01 to 0.39) (see Figure 2). Compared to males, females reported significantly higher MFQ scores in the LL (B = .94, SE = .25, *p* = 0.0001) and LS (B = .54, SE = .24, *p* = 0.03) groups but not in the SS group (B = .17, SE = .32, *p* = 0.60).

**Figure 3:**
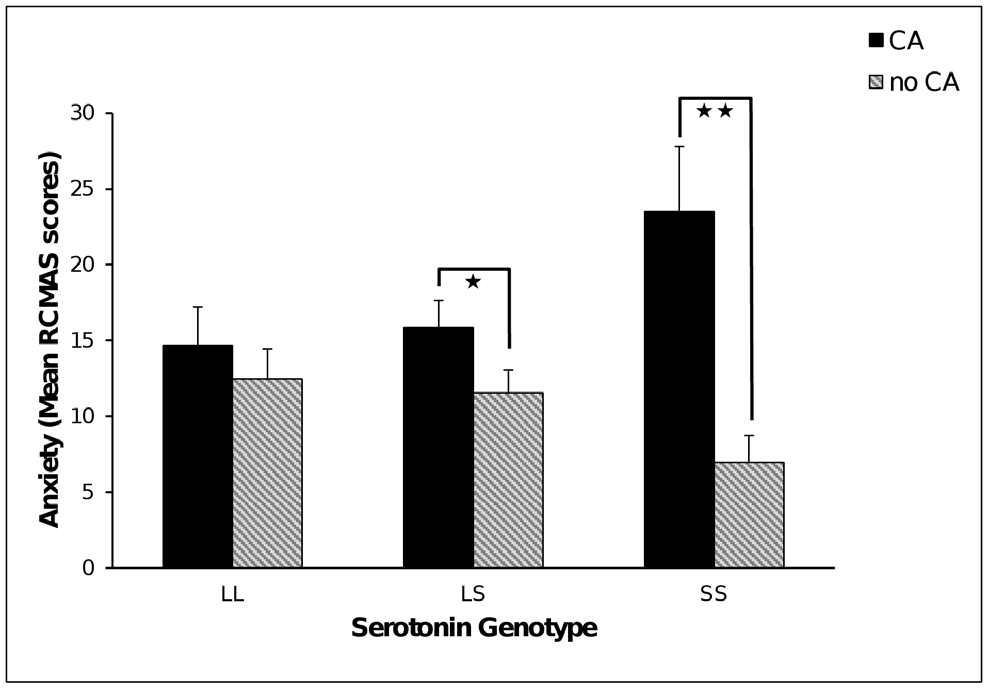
Mean scores of self-reported anxiety symptoms by gene variants in *5-HTTLPR* and early childhood adversities (CA). Error bars represent ±1 SE of the mean. * *p*<0.05; ** *p*<0.001.

We also found a significant *5-HTTLPR*×CA interaction on self-reported anxiety scores (RCMAS; Δχ^2^ = 8.03, df = 2, *p* = 0.02) that was similar in pattern to that revealed on MFQ scores (see Figure 3). CA was significantly related to higher anxiety in the SS (B = 2.10, SE = .53, *p*<0.0001, *ƒ^2^* = 0.66, 95%CI 0.20 to 1.69) and LS (B = .89, SE = .53, *p* = 0.005, *ƒ^2^* = 0.17, 95%CI 0.01 to 0.38) groups but not the LL group (B = .25, SE = .40, *p* = 0.53, *ƒ^2^* = 0.20, 95%CI 0.01 to 0.46). Females reported higher anxiety scores in the LL (B = 1.41, SE = .40, *p* = 0.0004) and LS (B = .940, SE = .31, *p* = 0.002) but not SS groups (B = .61, SE = .51, *p* = 0.23).

### Attentional Bias

**Figure 4:**
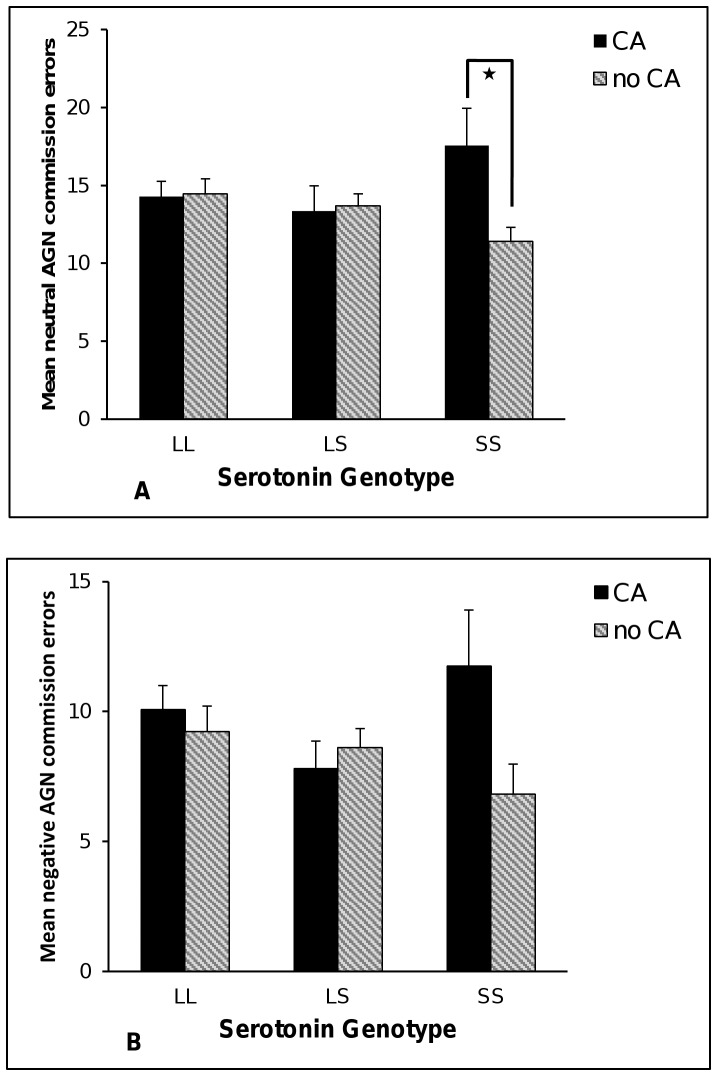
Mean number of emotionally valent neutral and negative commission errors by gene variants in *5-HTTLPR* and early childhood adversities (CA). Error bars represent ±1 SE of the mean. * *p*<0.05.

There were no main effects of *5-HTTLPR* or CA on AGN commission errors. There were, however, significant *5-HTTLPR*×CA interactions on negative (Δχ^2^ = 6.23, df = 2, *p* = 0.04) and neutral (Δχ^2^ = 6.12, df = 2, *p* = 0.05) (see Figure 4) but not positive (Δχ^2^ = 1.75 df = 2, *p* = 0.42) commission errors. Follow up comparisons using the Holm correction showed that CA was significantly related to more errors on the task for neutral (B = .61, SE = .25, *p* = 0.01, *ƒ^2^* = 0.33, 95%CI 0.00 to 0.65) but not negative word conditions (B = .70, SE = .34, *p* = 0.04, *ƒ^2^* = 0.28, 95%CI 0.02 to 0.72) for the SS gene group only. By comparison, CA was not related to error-making, before or after the Holm correction, in the negative condition for the LL (B = .12, SE = .22, *p* = 0.59, *ƒ^2^* = 0.13, 95%CI −0.01 to 0.31) or LS groups (B = −.28, SE = .23, *p* = 0.22, *ƒ^2^* = 0.12, 95%CI 0.00 to 0.28), or in the neutral condition for the LL (B = −.06, SE = .18, *p* = 0.75, *ƒ^2^* = 0.11, 95%CI −0.02 to 0.27) or LS groups (B = −.03, SE = .18, *p* = 0.88, *ƒ^2^* = 0.03, 95%CI −0.03 to 0.11). These results are all adjusted for the effects of IQ. We additionally tested for *5-HTTLPR*×CA interaction effects on AGN reaction times as a comparison. There were no significant effects for neutral, negative or positive conditions (see Table S3).

### Response to Negative Feedback

There were no main effects of *5-HTTLPR* or CA on any PRT variables except that CA was associated with more stage 2 switching (B = .46, SE = .20, *p* = 0.02, IRR = 1.59). There was, however, a significant *5-HTTLPR*×CA interaction on errors made before reaching task criterion in stage 1 (Wald = 11.16, df = 2, *p* = 0.004) (see Figure 4A). In the SS group CA was significantly related to the number of errors made to reach criterion (B = 1.78, SE = .43, *p*<0.0001, IRR = 5.93). This was contrasted with null effects in the LL (B = .11, SE = .43, *p* = 0.79, IRR = 1.12) and LS groups (B = −.16, SE = .48, *p* = . 0.75, IRR = 0.86). There was also a significant interaction in stage 2 (Wald = 6.23, df = 2, *p* = 0.04), where increased errors were associated with CA in the SS (B = .65, SE = .23, *p* = 0.004, IRR = 1.91) *and* LL groups (B = .47, SE = .16, *p* = 0.004, IRR = 1.60) when compared with the LS group (B = .01, SE = .17, *p* = 0.94, IRR = 1.01). These results are all adjusted for the effects of IQ.

**Figure 5:**
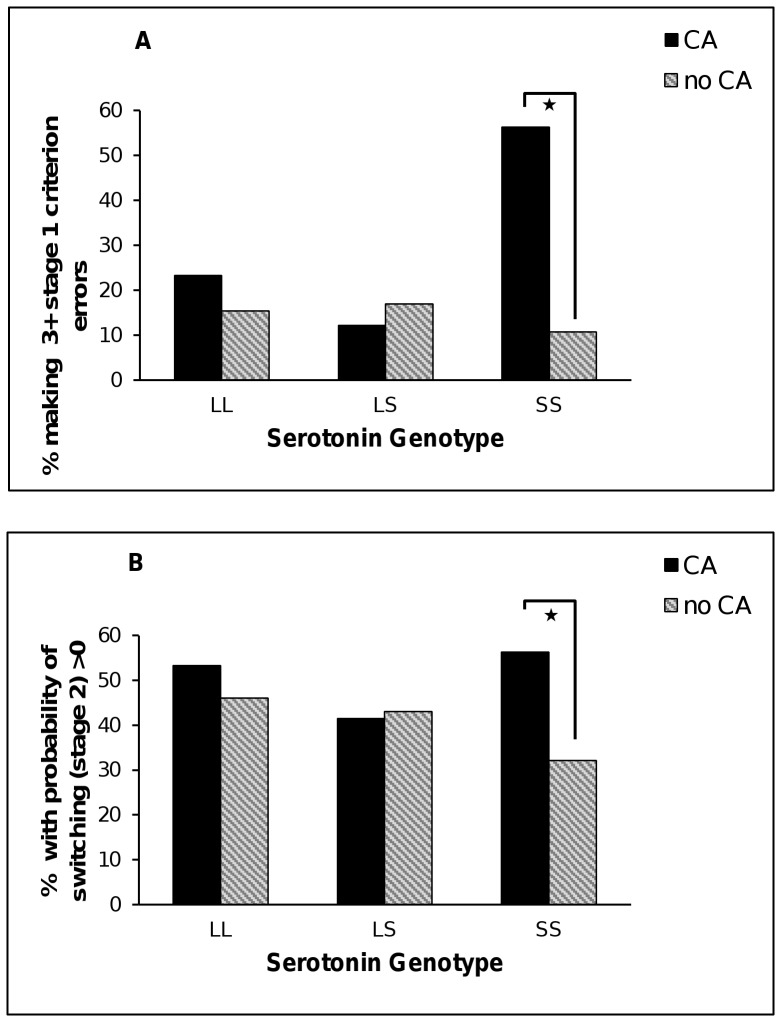
PRT results by gene variants in *5-HTTLPR* and early childhood adversities (CA). Panel A shows the proportion of participants making 3+ errors to reach task criterion in stage 1 in response to negative feedback. Panel B shows the probability of switching in response to negative feedback in stage 2. * *p*<0.01.

There was a non-significant trend for *5-HTTLPR*×CA interaction on the probability of switching response in the face of negative feedback in stage 1 (Wald = 5.36, df = 2, p = 0.07) and the pattern of data suggested that CA was significantly associated with switching in the SS group (B = 1.24, SE = .46, *p* = 0.007, IRR = 3.46) but not in the LL (B = .17, SE = .48, *p* = 0.72, IRR = 1.19) or LS groups (B = −.10, SE = .37, *p* = 0.79, IRR = 0.91). In stage 2 there was a significant *5-HTTLPR*×CA interaction (Wald = 7.56, df = 2, *p* = 0.02), whereby CA was associated with switching in the SS group (B = 1.13, SE = .38, *p* = 0.003, IRR = 3.09) but not in the LS (B = −.13, SE = .28, *p* = 0.64, IRR = 0.88) or LL groups (B = .52, SE = .31, *p* = 0.10, IRR = 1.68) (see Figure 5B).

### Paired Associates Learning

There were no main effects of *5-HTTLPR* or CA on the PAL test, or any interaction (Wald = 1.35, df = 2, *p* = 0.510). CA was not significantly related to memory in the SS (B = −.01, SE = .27, *p* = 0.96, IRR = 0.99), LS (B = −.01, SE = .16, *p* = 0.91, IRR = 0.99) or LL gene groups (B = −.31, SE = .22, *p* = 0.19, IRR = 0.73). These results are all adjusted for the effects of IQ.

### Emotional Disorder

Females were more likely to receive a diagnosis of an emotional disorder (anxiety or depressive disorder) (OR = 3.51, *p* = 0.002, 95%CI, 1.56 to 7.87). We also found significant associations between the AGN commission errors for neutral (OR = 1.82, *p* = 0.006, 95%CI, 1.19 to 2.78) and negative (OR = 1.46, *p* = 0.02, 95%CI, 1.04 to 2.02), but not positive valenced words (OR = 1.35, *p* = 0.09, 95%CI, .96 to 1.89) and any emotional disorder at 17 years. The effects for neutral and negative errors remained significant after the Holm correction. Similarly, there was an association between response to negative feedback and disorder for stage 1 errors (OR = 1.10, *p* = 0.04, 95%CI, 1.05 to 1.21; but not stage 2 errors, OR = 1.03, *p* = 0.34, 95%CI, .96 to 1.11) and the probability of switching in stage 2 (OR = 1.47, *p* = 0.02, 95%CI, 1.01 to 2.05; but not in stage 1 OR = 1.27, *p* = 0.11, 95%CI, .95 to 1.71). These effects were not however significant after applying the Holm correction.

Conversely, there was no significant association between simple memory (PAL) and disorder (OR = 1.04, *p* = 0.12, 95%CI, .99 to 1.09). These results are all adjusted for the effects of sex.

The results of the triallelic genotyping analysis are shown in Table S4. There was a similar pattern in these analyses in that the majority of associations between CA and the dependent variables were significant in the S′S′ or S″S″ groups only. In the S″S″ analysis the interaction effects were broadly similar, with the exception for RCMAS (*p* = 0.06), PRT stage 2 errors stage (*p* = 0.10) and PRT stage 2 switching (*p* = 0.06). Re-allocating the L_G_ allele had the effect of removing the majority of significant interactions with the exception of MFQ (*p* = 0.01).

## Discussion

In a large sample of adolescents, we found that *5-HTTLPR* in combination with exposure to CA up to the age of 6 years explained individual differences in self-reported depression and anxiety symptoms, which is consistent with prior research [17], [18], [66], [74]. Results of recent G×E meta-analyses on depression have been equivocal [19], [20], [75] and there is some evidence to suggest that methodological differences between studies may partly explain this discrepancy. Using a rigorous interview method to ascertain the nature of the individual's early environmental experience rather than questionnaires may enable the better detection of G×E effects [51], [52]. Consistent with this suggestion, the positive findings in the present study may have in part been revealed by the robust measure of CA.

The *5-HTTLPR*×CA interactions for the self-report depression and anxiety scores were driven by being homozygous for the S allele. Within this group, however, was a further subset of raised scores for those who had experienced CA but lowered scores for those with no history of CA (see Figures 2 and 3). These results provide indirect support for a differential susceptibility [25] and not just a diathesis-stress effect. Caution is required however as this is not a direct test of susceptibility as the absence of adversity may not equate to the presence of a positive environment. The finding is consistent however with Fox et al. [31] who showed that attentional biases towards emotional stimuli were increased by *5-HTTLPR* SS genotype, by both negative and positive attentional bias induction procedures.

Turning to the neuropsychological test results, we demonstrated that SS carriers exposed to CA displayed more attentional bias on neutral but not positive stimuli conditions on the AGN task. This finding is consistent with prior research in the literature that SS carriers exposed to adversities have greater difficulty in classifying ambiguously valenced stimuli [45], [76], [77]. There was also a suggestion in the data that a similar interaction was operating on negative words, which extends previous research with *5-HTTLPR* S homozygote children who were poor at recognising fearful faces [38], by showing that the social environment interacts with *5-HTTLPR*. However, this latter effect did not remain significant after corrections for multiple comparisons. The same pattern of interaction was found on the PRT task where response to ambiguous negative feedback led to significantly more errors and an increased likelihood of switching responses.

Finally, we found a positive association between the aforementioned differences in AGN and PRT task performance and any emotional disorder occurring an average of 1 year after testing. These findings are consistent with current cognitive models of emotional psychopathology that argue that such cognitive and emotional processing lies intermediate between G×E and disorder [33]. Taking these findings together, the results lend support to our hypothesis that aberrant cognitive processing of emotion occurs in adolescents who are SS carriers and were exposed to CA nearly a decade earlier. This may however reflect only the negative pole of indviduals who carry the SS variant and the trend in these results may reveal that SS carriers are more susceptible to their social environments both good as well as bad. From the psychopathology perspective, however, these findings clearly support the hypothesis that there is an intermediate phenotype for depression and anxiety indexed by aberrant information processing present in the adolescent years [33]. The study was not designed nor powered to undertake longitudinal analyses so we cannot fully test the temporal links between all variables measured. Thus the results presented here cannot fully test the hypothesis, but provide strong proof of principle for it. Recent evidence that polymorphisms in *5-HTTLPR* predict the magnitude of experimentally induced attentional bias to emotional stimuli [31] provides further support for the findings reported in this paper.

In order to explain the apparent lack of association between pathology in the brain and clinical symptoms [78], theoretical models of a third factor, ‘cognitive reserve’, have been developed in psychopathology research. Such models may be passive, focusing on the ‘hardware’ of the brain, and imply that a high neural capacity or threshold can act as a protective factor against pathology. Active models refer to the cognitive processing or ‘software’ of the brain which may be employed as a compensatory mechanism, again buffering against the negative effects of pathology. Active models are often indexed by markers such as IQ [79]. Crucially, the interaction effects on the cognitive and emotional variables in the present study remained significant when IQ was taken into account. Finally, as hypothesised, we demonstrated no such *5-HTTLPR*×CA effects on a visuo-spatial memory task that did not include any explicit emotional content. Together these findings suggest that the low cognitive reserve hypothesis of psychopathology [80] is not of itself sufficient to explain the *5-HTTLPR*×CA interaction effects in the current findings.

From the findings in the current cross-sectional study our *hypothesised* developmental model starts with the interaction between allelic variation in *5-HTTLPR* and CA and leads to cognitive and emotional processing style that may lead some individuals to develop one of a range of emotional disorders. Nevertheless, an equally plausible model is one where gene-environment interplay leads directly to an emotional disorder and then subsequently to differences in cognitive and emotional processing. But because the latter is associated with emotional disorders, a third reciprocal model is possible, where a cycle of emotional psychopathology and cognitive emotional processing exacerbate one another. Further research should test these possibilities.

Interestingly and perhaps regardless of which *5-HTTLPR*×CA process is mechanistic, the current findings point to a transdiagnostic intermediate cognitive phenotype for a range of anxiety and depressive disorders rather than one specific to a particular clinical typology. Our secondary triallelic analyses were somewhat less clear cut. With the L_G_ allele removed and thereby testing for effects between groups with the highest and lowest transcriptional activity, the results were similar in pattern but weaker overall. When the L_G_ was re-allocated to either the LS or SS biallelic groups however the only significant interaction found was for MFQ. As noted the assumption is that the triallelic classification is more sensitive through reallocation of the L_G_ SNP as it is deemed functionally equivalent to the S allele in *5-HTTLPR* [5]. We noted in the introduction that these findings have been inconsistent [8], [9] and it is also unclear what the impact of the rs25531 A>G SNP has on the S allele [81]. One explanation for the lack of significance in the triallelic (S′S′) results in the present study may be that the L_G_ allele acts to dilute the impact in the interaction with CA, thereby rendering the individuals less susceptible or sensitive than their SS counterparts. This view is consistent with the findings of Hu et al. [7] who found that although levels of mRNA expression between L_G_ L_G_ and SS groups were not statistically different from one another, the latter displayed the lowest levels in absolute terms. The current findings removing the L_G_ allele may be reflecting a more sensitive distinction with regard to the putative interactions with the social environment. Further research is required to test the interaction hypothesis using both genotyping variations.

### Strengths and limitations

A main strength of the current study was the use of a detailed interview method (CAMEEI) with an informed respondent to capture early childhood adversities [53]. The study had, however, low statistical power overall due to the natural low prevalence rates for adolescents with CA also possessing short copies of the *5-HTTLPR* allele. In particular, this precluded the examination of any *5-HTTLPR*×CA effects on the presence of emotional disorder (see Table S2). Therefore, type II errors may have occurred in the analysis. Conclusions should therefore still be drawn with caution and future replication studies should endeavour to use larger sample sizes to resolve these issues.

Additionally it is possible that past episodes of depression or other mood related psychopathologies in childhood could have resulted in permanent residual effects leading to cognitive differences [82]. Nevertheless, should any traces of previous episodes of depression (physiological or psychological) exist, they could either be due to prior psychiatric symptomatology embedded and measured in the depression and anxiety symptoms or to the triggers that are the primary cause of such symptoms [83], i.e. genes and the environment. The results of this study suggest that both symptoms of anxiety and depression and cognitive and emotional processing are affected by the *5-HTTLPR*×CA interaction whilst being only weakly related to one another. Similarly, whether the current results are truly a serotonergic mediated psychological precursor for individual differences in emotion processing [3], [11] remains to be determined by prospective research.

Overall we suggest these findings from a community ascertained population provide evidence that aberrant cognitive and emotional processing is a testable transdiagnostic intermediate marker for emerging emotional disorders in adolescents and young adults. Finally we do not underestimate the neural complexities that may underlie the deficits in cognitive and emotional processing revealed in this study. It is important to recall that behavioural outputs occur as an end stage product of multiple neurobiological pathways not measured here [84]. Neither do we make claims that we have in any way refuted a genetic association with early adversities or cognitive and emotional processes using other gene variants [85]. In a recent meta-analysis on the association between *5-HTTLPR* polymorphisms and unipolar depression, a small but significant association was found (OR = 1.16) for a recessive (i.e. SS versus L+) but not dominant model (i.e. S versus LL). The findings of the meta-analysis first show that the likely influence of single genes in explaining psychopathology is going to be small [86]. Secondly, these findings reinforce the necessity for taking into account the social environment. Lastly, they suggest that a recessive model may be most likely to reveal *5-HTTLPR*×CA interaction effects on depressive psychopathology. This hypothesis should be further tested in psychiatric studies and also requires more genetic work. For example recent evidence suggests that *5-HTTLPR* may contain further functional alleles [10].

In conclusion, this is one of the first studies to reveal a putative gene-environment basis for individual differences in a cognitive-emotionally mediated pathway as a transdiagnostic intermediate phenotype to psychopathology that requires replication and validation in future longitudinal designs.

## Supporting Information

Table S1 Alignment to Gaussian distribution before and after square root transformation
(DOCX)

Table S2 Sample Characteristics by 5-HTTLPR and CA groups
(DOCX)

Table S3 AGN reaction times (RT) by 5-HTTLPR and CA groups
(DOCX)

Table S4 Triallelic 5-HTTLPR v CA interaction analysis
(DOCX)

File S1 Supporting information
(DOCX)
