# Supplementary material for: 5-HTTLPR and Early Childhood Adversities Moderate Cognitive and Emotional Processing in Adolescence
Source: PLoS One. 2012 Nov 28;7(11):e48482. doi: 10.1371/journal.pone.0048482 (PMC3509124; doi:10.1371/journal.pone.0048482)
Supplement: Table S1 — Alignment to Gaussian distribution before and after square root transformation (DOCX) [file pone.0048482.s001.docx]

| **Table S1.** *Alignment to Gaussian distribution before and after square root transformation* | | |
| --- | --- | --- |
| Variable | Untransformed distribution | Square root transformation |
| AGN positive | χ^2^ = 30.39, *p* < .001 | χ^2^ = 0.13, *p* = .94 |
| AGN neutral | χ^2^ = 24.81, *p* < .001 | χ^2^ = 9.07, *p* = .01 |
| AGN negative | χ^2^ = 48.44, *p* < .001 | χ^2^ = 1.24, *p* = .54 |
| MFQ | χ^2^ = 37.09, *p* < .001 | χ^2^ = 1.24, *p* = .46 |
| RCMAS | χ^2^ = 46.48, *p* < .001 | χ^2^ = 1.24, *p* = .46 |

*Note.* The χ^2^ statistic and associated *p –* value here refers how closely the distributions of variables fit to a Gaussian distribution. AGN = Affective Go/No-Go; MFQ = Mood and Feelings Questionnaire; RCMAS = Revised Children’s Manifest Anxiety Scale.
