# Supplementary material for: 5-HTTLPR and Early Childhood Adversities Moderate Cognitive and Emotional Processing in Adolescence
Source: PLoS One. 2012 Nov 28;7(11):e48482. doi: 10.1371/journal.pone.0048482 (PMC3509124; doi:10.1371/journal.pone.0048482)
Supplement: Table S2 — Sample Characteristics by 5-HTTLPR and CA groups (DOCX) [file pone.0048482.s002.docx]

| **Table S2.** *Sample Characteristics by 5-HTTLPR and CA groups* | | | | | | | | | | | | |
| --- | --- | --- | --- | --- | --- | --- | --- | --- | --- | --- | --- | --- |
|  | LL | | | | LS | | | | SS | | | |
|  | N=87 | | | | N=107 | | | | N=44 | | | |
|  | CA | | No CA | | CA | | No CA | | CA | | No CA | |
|  | N=31 (36%) | | N=56 (64%) | | N=41 (38%) | | N=66 (62%) | | N=16 (36%) | | N=28 (64%) | |
|  | Mean/N | *SD*/% | Mean/N | *SD*/% | Mean/N | *SD*/% | Mean/N | *SD*/% | Mean/N | *SD*/% | Mean/N | *SD*/% |
| Age | 16.4 | (0.5) | 16.3 | (0.4) | 16.3 | (0.4) | 16.4 | (0.4) | 16.9 | (0.8) | 17.1 | (0.9) |
| Sex Female, N (%) | 20 | (65) | 29 | (52) | 14 | (34) | 28 | (42) | 13 | (76.5) | 14 | (50) |
| IQ | 101.7 | (14.1) | 108.7 | (17.7) | 107.9 | (15.0) | 109.9 | (14.6) | 97.9 | (16.3) | 105.3 | (13.5) |
| MFQ | 14.9 | (9.4) | 14.0 | (9.1) | 14.4 | (10.2) | 9.7 | (6.4) | 17.6 | (9.7) | 7.3 | (5.7) |
| RCMAS | 14.7 | (13.4) | 12.5 | (12.7) | 15.8 | (10.3) | 11.5 | (11.4) | 23.5 | (14.9) | 7.0 | (8.6) |
| Emotional disorder† | 7 | (24) | 9 | (17) | 5 | (14) | 6 | (9) | 5 | (33) | 3 | (11) |

*Note.* IQ = Weschler intelligence scale for children (III); MFQ = Mood and Feelings Questionnaire; RCMAS = Revised Children’s Manifest Anxiety Scale. † data were available for LL + CA (N=29), LL - CA (N=54); LS + CA (N=37), LS - CA (N=66); SS + CA (N=15), SS - CA (N=28).
