# Supplementary material for: 5-HTTLPR and Early Childhood Adversities Moderate Cognitive and Emotional Processing in Adolescence
Source: PLoS One. 2012 Nov 28;7(11):e48482. doi: 10.1371/journal.pone.0048482 (PMC3509124; doi:10.1371/journal.pone.0048482)
Supplement: Table S3 — AGN reaction times (RT) by 5-HTTLPR and CA groups (DOCX) [file pone.0048482.s003.docx]

| **Table S3.** *AGN reaction times (RT) by 5-HTTLPR and CA groups* | | | | | | | | | | | | |
| --- | --- | --- | --- | --- | --- | --- | --- | --- | --- | --- | --- | --- |
|  | LL | | | | LS | | | | SS | | | |
|  | CA | | No CA | | CA | | No CA | | CA | | No CA | |
|  | Mean | *SD* | Mean | *SD* | Mean | *SD* | Mean | *SD* | Mean | *SD* | Mean | *SD* |
| AGN neutral RT | 521.89 | (106.15) | 526.83 | (86.93 ) | 537.05 | (101.78) | 526.21 | (94.94) | 506.83 | (123.35 ) | 537.09 | (73.71) |
| AGN negative RT | 476.73 | (77.96) | 485.74 | (65.12) | 490.40 | (68.08) | 492.80 | (79.20) | 482.63 | (89.58) | 493.23 | (65.02) |
| AGN positive RT | 464.40 | (67.99) | 474.97 | (56.66 ) | 480.42 | (77.43) | 475.32 | (76.33) | 469.99 | (93.78) | 472.73 | (54.41) |

*Note.* AGN = Affective Go/No-Go (AGN) task.
