# Supplementary material for: 5-HTTLPR and Early Childhood Adversities Moderate Cognitive and Emotional Processing in Adolescence
Source: PLoS One. 2012 Nov 28;7(11):e48482. doi: 10.1371/journal.pone.0048482 (PMC3509124; doi:10.1371/journal.pone.0048482)
Supplement: Table S4 — Triallelic 5-HTTLPR v CA interaction analysis (DOCX) [file pone.0048482.s004.docx]

| **Table S4.**  *Triallelic 5-HTTLPR v CA interaction analysis* | | | | | | | | |
| --- | --- | --- | --- | --- | --- | --- | --- | --- |
|  | L′L′ | L′S′ | S′S′ | Interaction | L′′L′′ | L′′S′′ | S′′S′′ | Interaction |
| MFQ | 0.251 (0.358) | 0.428 (0.087) | **1.371 (<0.001)** | **0.0140** | 0.251 (0.358) | **0.679 (0.009)** | **1.545 (<0.001)** | **0.0099** |
| RCMAS | 0.474 (0.317) | **0.804 (0.010)** | **1.622 (<0.001)** | 0.1781 | 0.474 (0.317) | **0.987 (0.003)** | **2.163 (<0.001)** | 0.0612 |
| AGN neutral | -0.095 (0.611) | 0.008 (0.968) | **0.420 (0.043)** | 0.1489 | -0.095 (0.611) | 0.023 (0.915) | **0.631 (0.016)** | **0.048** |
| AGN negative | 0.126 (0.578) | -0.211(0.400) | 0.469 ( 0.094) | 0.1942 | 0.126 (0.578) | -0.262 (0.319) | **0.781 (0.027)** | **0.049** |
| AGN positive | 0.233 (0.301) | 0.115 (0.608) | 0.261 (0.295) | 0.804 | 0.233 (0.301) | 0.107 (0.655) | 0.427 (0.180) | 0.5398 |
| PRT s1 errors | 0.176 (0.707) | 0.112 (0.802) | **0.948 (0.093)** | 0.4631 | 0.174 (0.710) | 0.149 (0.752) | **1.854 (<0.001)** | **0.0188** |
| PRT s2 errors | **0.419 (0.023)** | 0.083 (0.625) | **0.498 (0.011)** | 0.2151 | **0.419 (0.024)** | 0.008 (0.966) | **0.562 (0.010)** | 0.1046 |
| PRT s1 switching | 0.165 (0.770) | 0.329 (0.332) | 0.428 (0.293) | 0.9310 | 0.164 (0.771) | 0.249 (0.512) | **1.219 (0.016)** | 0.2435 |
| PRT s2 switching | 0.482 (0.202) | 0.114 (0.635) | **0.774 (0.036)** | 0.2977 | 0.467 (0.219) | -0.038 (0.889) | **1.116 (0.008)** | 0.0627 |
| PAL errors | -0.264 (0.291) | -0.040 (0.812) | -0.136 (0.586) | 0.7534 | -0.265 (0.290) | 0.042 (0.817) | -0.030 (0.911) | 0.993 |

*Note.* Triallelic genotype groups (L′L′ (n=70), L′S′ (n=106) and S′S′ (n=59)) were derived in the following way: L_A_L_A_ = L′L′; L_A_L_G_ & L_A_S = L′S′; L_G_S & SS = S′S′. Genotype groupings with the L_G_ allele excluded (L′′L′′ (n=70), L′′S′′ (n=90) and S′′S′′ (n=43)) were derived in the following way: L_A_L_A_ = L′′L′′; L_A_S = L′′S′′; SS = S′′S′′. The coefficients refer to the association between CA and the dependent variable indicated in the row. Figures in parentheses and under the heading ‘Interaction’ are p-values. Values in Bold are significant (*p* < 0.05).
