# Supplementary material for: 5-HTTLPR and Early Childhood Adversities Moderate Cognitive and Emotional Processing in Adolescence
Source: PLoS One. 2012 Nov 28;7(11):e48482. doi: 10.1371/journal.pone.0048482 (PMC3509124; doi:10.1371/journal.pone.0048482)
Supplement: File S1 — Supporting information (DOCX) [file pone.0048482.s005.docx]

**Supporting Information**

The comparison of the distributions of cognitive and emotional variables before and after transformation are shown in Table S1. The distributions of the variables are vastly improved after transformation. In the inferential analyses we also used a maximum likelihood mean and variance adjusted estimator (MLMV) which is robust to non-normality.

The sample characteristics broken down by childhood adversities (CA) and *5-HTTLPR* genotype groups are shown in Table S2. The comparison of AGN reaction times (RT) is illustrated in Table S3. There were no significant *5-HTTLPR* x CA interactions for neutral (*p =* 0.493) negative (*p =* 0.955) or positive (*p =* 0.773) conditions.

As detailed in the main text, there has been some uncertainty as to the functional significance of an A>G SNP (rs25531) in the long allele (L) for *5-HTTLPR* [1]. It was originally thought that the short (S) allele possessed less transcriptional efficiency than the L allele [2]. More recently, it has been suggested that the *5-HTTLPR* L allele is not homogeneous in its functional activity and that the L_A_ variant of rs25531 only has higher efficiency; the L_G_ functions more like the S allele [3]. However, some studies have not supported this view [4,5]. In light of the equivocal findings we carried out further exploratory analyses on the rs25531 SNP.

We reanalysed our data additionally genotyping for rs25531 and assessed the pattern in the data by genotype and childhood adversities (CA) groups. The triallelic genotype groupings (L′L′ (n=70), L′S′ (n=106) and S′S′ (n=59)) were derived in the following way: L_A_L_A_ = L′L′; L_A_L_G_ & L_A_S = L′S′; L_G_S & SS = S′S′ after Parsey et al. [5]. Given the uncertainty over the functional significance of the L_G_ allele we performed a parallel analysis with the L_A_ allele only. The results are presented in Table S4 below.

We found a broadly similar pattern of results in that the hypothesised associations between CA and the cognitive and emotional variables were largely seen in the ‘SS’ groups. However, in the triallelic analysis, the majority of interaction effects were non-significant, with the exception of MFQ. Removing the L_G_ allele from the analysis produced much closer results to the original biallelic results. Collectively the findings suggest that the interaction signal between *5-HTTLPR* and CA on cognitive and emotional variables is more readily detected using S rather than the L_G_ carriers. This is discussed in the main text and we propose that susceptibility to the environment may be proportional to transcriptional activity indexed by L_A_>L_G_>S. We suggest that L_G_/S carriers may be insufficiently sensitive to the environmental adversities and this suppresses the distinction between LL and SS groups in the analyses. This is hypothesis forming and requires further detailed work. The results presented in Table S3 suggest that this is plausible. For example, the effect of CA on PRT s1errors in the S′′ S′′ group (L_A_ only) is approximately twice that found in the S′S′ (triallelic) group.

References

1. Wendland JR, Martin BJ, Kruse MR, Lesch KP, Murphy DL (2006) Simultaneous genotyping of four functional loci of human SLC6A4, with a reappraisal of 5-HTTLPR and rs25531. Molecular Psychiatry 11: 224-226.

2. Lesch KP, Bengel D, Heils A, Sabol SZ, Greenberg BD, et al. (1996) Association of anxiety-related traits with a polymorphism in the serotonin transporter gene regulatory region. Science 274: 1527-1531.

3. Hu XZ, Lipsky RH, Zhu G, Akhtar LA, Taubman J, et al. (2006) Serotonin transporter promoter gain-of-function genotypes are linked to obsessive-compulsive disorder. Am J Hum Genet 78: 815-826.

4. Martin J, Cleak J, Willis-Owen SA, Flint J, Shifman S (2007) Mapping regulatory variants for the serotonin transporter gene based on allelic expression imbalance. Molecular Psychiatry 12: 421-422.

5. Parsey RV, Hastings RS, Oquendo MA, Hu X, Goldman D, et al. (2006) Effect of a triallelic functional polymorphism of the serotonin-transporter-linked promoter region on expression of serotonin transporter in the human brain. American Journal of Psychiatry 163: 48-51.
